# Effect of antiplatelet therapy on the incidence, prognosis, and rebleeding of intracerebral hemorrhage

**DOI:** 10.1111/cns.14175

**Published:** 2023-03-21

**Authors:** Yunjie Li, Xia Liu, Shiling Chen, Jingyi Wang, Chao Pan, Gaigai Li, Zhouping Tang

**Affiliations:** ^1^ Department of Neurology Tongji Hospital, Tongji Medical College, Huazhong University of Science and Technology Wuhan China

**Keywords:** antiplatelet drugs, antiplatelet therapy, hematoma expansion, intracerebral hemorrhage, recurrent ICH

## Abstract

**Objective:**

Antiplatelet medications are increasingly being used for primary and secondary prevention of ischemic attacks owing to the increasing prevalence of ischemic stroke occurrences. Currently, many patients receive antiplatelet therapy (APT) to prevent thromboembolic events. However, long‐term use of APT might also lead to an increased occurrence of intracerebral hemorrhage (ICH) and affect the prognosis of patients with ICH. Furthermore, some research suggest that restarting APT for patients who have previously experienced ICH may result in rebleeding events. The precise relationship between APT and ICH remains unknown.

**Methods:**

We searched PubMed for the most recent related literature and summarized the findings from various studies. The search terms included “antiplatelet,” “intracerebral hemorrhage,” “cerebral microbleeds,” “hematoma expansion,” “recurrent,” and “reinitiate.” Clinical studies involving human subjects were ultimately included and interpreted in this review, and animal studies were not discussed.

**Results:**

When individuals are administered APT, the risk of thrombotic events should be weighted against the risk of bleeding. In general, for some patients’ concomitant with risk factors of thrombotic events, the advantages of antiplatelet medication may outweigh the inherent risk of rebleeding. However, the use of antiplatelet medications for other patients with a higher risk of bleeding should be carefully evaluated and closely monitored. In the future, a quantifiable system for assessing thrombotic risk and bleeding risk will be necessary. After evaluation, the appropriate time to restart APT for ICH patients should be determined to prevent underlying ischemic stroke events. According to the present study results and expert experience, most patients now restart APT at around 1 week following the onset of ICH. Nevertheless, the precise time to restart APT should be chosen on a case‐by‐case basis as per the patient's risk of embolic events and recurrent bleeding. More compelling evidence‐based medicine evidence is needed in the future.

**Conclusion:**

This review thoroughly discusses the relationship between APT and the development of ICH, the impact of APT on the course and prognosis of ICH patients, and the factors influencing the decision to restart APT after ICH. However, different studies' conclusions are inconsistent due to the differences in quality control. To support future clinical decisions, more large‐scale randomized controlled trials are required.

## Graphical abstract

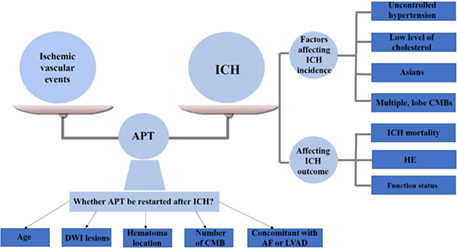

## 1 INTRODUCTION

The activation and aggregation of platelet are the root cause of arterial thrombosis. Antiplatelet drugs have been reported to reversibly or irreversibly inhibit platelet aggregation and reduce blood coagulation's ability to prevent thrombosis and vascular embolization events.^1^ Some of the commonly used antiplatelet drugs include aspirin (inhibits thromboxane‐A2 production), clopidogrel and ticagrelor (inhibit adenosine diphosphate receptor on platelet membrane), and dipyridamole (inhibits adenosine uptake and the enzyme cyclic guanine monophosphate phosphodiesterase).^2^ Dual antiplatelet therapy (APT), which involves the use of two antiplatelet drugs with different mechanisms, was previously used for patients with myocardial infarction who did not have antithrombotic contraindications. Later, dual APT began to be recommended for patients who have had drug‐eluting and bare‐metal stents placed.^3^, ^4^ The early treatment with aspirin plus extended‐release dipyridamole for transient ischemic attack or ischemic stroke within 24 h of symptom onset (EARLY) trial subsequently demonstrated that combination therapy with aspirin and dipyridamole is effective for the secondary prevention of cerebrovascular disease and transient ischemic attack, with no increased risk of bleeding compared with the use of aspirin alone.^5^ Following the publication of the Clopidogrel in High‐risk patients with Acute Non‐disabling Cerebrovascular Events (CHANCE) trial, combination therapy with aspirin and clopidogrel for 21 days was widely used in patients with acute cerebral infarction and transient ischemic attacks.^6^ The Platelet‐Oriented Inhibition in New Transient Ischemic Attack and Minor Ischemic Stroke (POINT) study reported that aspirin combined with clopidogrel for 90 days reduced the risk of major ischemic events (hazard ratio [HR] 0.75%, 95% confidence interval [CI], 0.59–0.95, *p* = 0.02) but increased the risk of major hemorrhage (HR 2.32; 95% CI 1.10–4.87, *p* = 0.02) compared with patients treated with aspirin alone.^7^ The CHANCE‐2 study found that ticagrelor plus aspirin was more effective than clopidogrel plus aspirin in preventing recurrent stroke in patients with mild ischemic stroke and transient ischemic attack carrying the CYP2C19‐inactivated allele.^8^ Multiple studies have demonstrated that combining dual antiplatelet drugs for 3 months can reduce recurrent ischemic events without increasing bleeding events in patients with severe intracranial macrovascular stenosis (70%–99%).^9^, ^10^, ^11^, ^12^ To prevent recurrent stroke events, The American Heart Association/American Stroke Association (AHA/ASA) for 2021 recommend that such patients should be treated with dual antiplatelet drugs for 3 months, followed by treatment with a single antiplatelet drug.^13^

**FIGURE 1:**
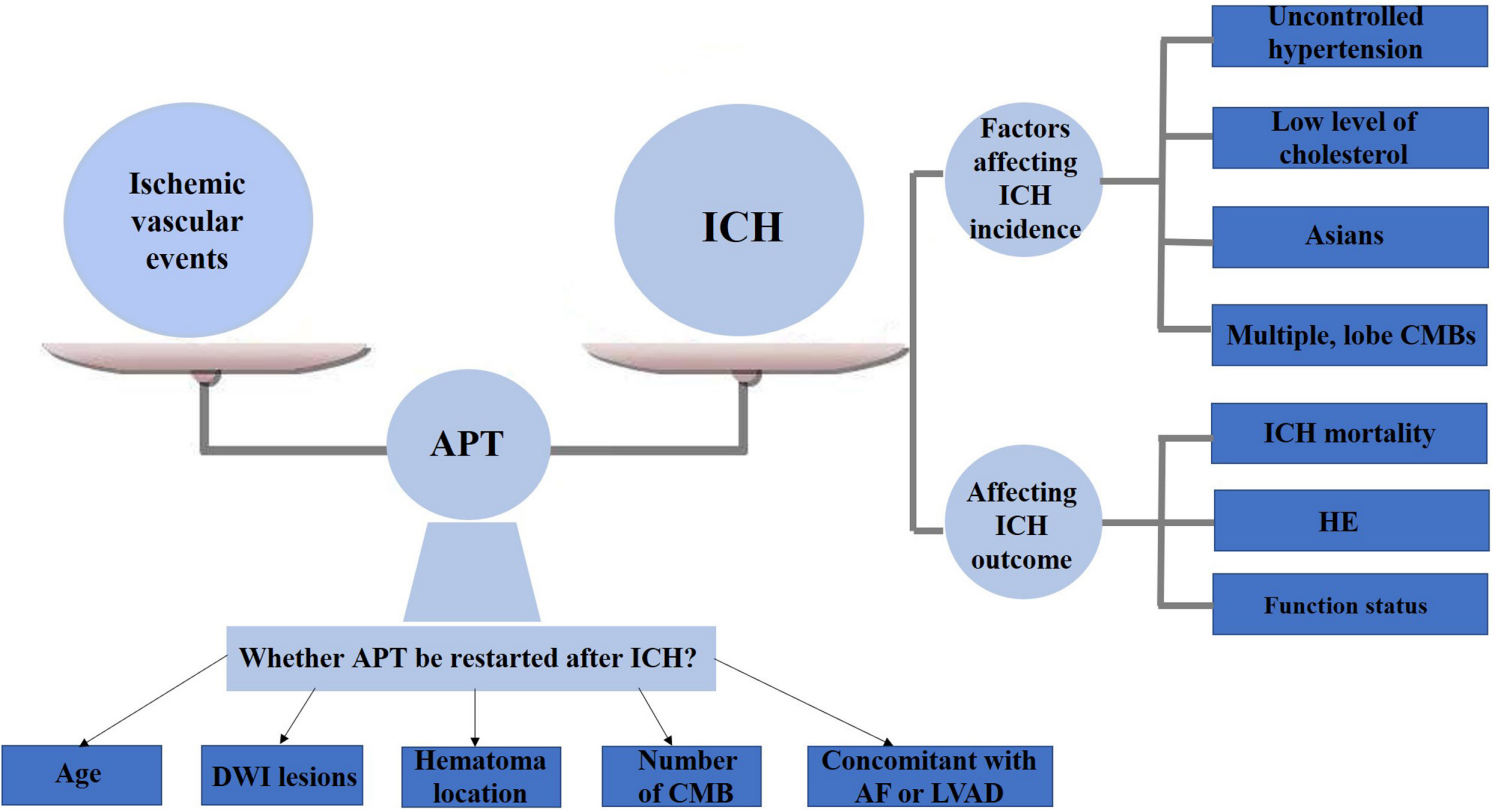
Effect of APT on ICH. APT, antiplatelet therapy; CMBs, cerebral microbleeds; DWI, diffusion‐weighted imaging; HE, hematoma expansion; ICH, intracerebral hemorrhage; LVAD, left ventricular assist devices.

Many studies have demonstrated that long‐term APT could reduce the frequency of ischemic cardiovascular and cerebral events. However, it might also cause an increased occurrence of intracerebral hemorrhage (ICH).^14^, ^15^ With an annual incidence of 10–30 per 100,000 persons, ICH is the second most common type of stroke after cerebral infarction.^16^ It has been reported that the mortality rate of patients with ICH is as high as 50% within 1 month of onset, and only less than 40% of patients eventually return to functional independence.^17^ Approximately 20%–30% of ICH patients were reported to be on APT.^18^ The benefits of APT in reducing the risk of ischemic events and the possibility of hemorrhagic events following APT need to be evaluated individually to develop a safe and effective dosing regimen. It is also unknown how antiplatelet aggregating medications affect the development and prognosis of ICH. Furthermore, it is important to consider whether APT should be restarted after ICH. To address the abovementioned unclear clinical issues, in this review, we summarize related studies and discuss the influence of APT on the incidence of ICH, the effects of prior APT on the development and prognosis of ICH, and whether to restart APT in patients with a history of ICH (Figure 1). We searched PubMed for the most recent related literature and summarized the findings from various studies. The search terms included “antiplatelet,” “intracerebral hemorrhage,” “cerebral microbleeds,” “hematoma expansion,” “recurrent,” and “reinitiate.” Clinical studies involving human subjects were ultimately included and interpreted in this review, and animal studies were not discussed.

## 2 EFFECT OF APT ON ICH OCCURRENCE

### 2.1 Influence of prior APT on the incidence of ICH

Antiplatelet drugs are frequently used to prevent acute cardiocerebrovascular attacks in individuals at a high risk of ischemic stroke. However, it remains unclear whether these drugs might increase the incidence of ICH events.^19^ According to some studies, APT may increase the incidence of ICH. For instance, He et al. found that among 55,462 individuals (asprin group 28,570 patients vs. control group [placebo] 26,892 patients; mean aspirin dose of 273 mg/day; duration 37 months in the aspirin group), the aspirin group had a higher ICH risk (12 per 10, 000 people, 95% CI 5–20, *p* < 0.001) than the control group.^14^ Another study found that the risk of ICH attacks caused by APT increased with age (odds ratio [OR] 1.05, 95% CI 1.03–1.07) and hypertension level (OR 1.86, 95% CI 1.22–2.85).^20^ The risk variables for intraoperative and postoperative rebleeding in ICH patients who received minimally invasive surgery were examined by Brouwers et al.; and the findings demonstrated that previous APT was a predictive marker for postoperative rebleeding.^21^ Hald et al. compared changes in the use of antiplatelet drugs and anticoagulants between 2005 and 2018 and found that the age‐ and sex‐matched incidence of ICH decreased from 33 per 10,000 person‐years in 2005 to 24 per 10,000 person‐years in 2018 (*p* < 0.001 for the trend) as the frequency of antiplatelet medication use reduced (24.7% in 2005 vs. 21.4% in 2018).^22^

While some researchers reported that APT did not affect ICH.^23^, ^24^ García‐Rodríguez et al. found that APT decreased the risk of subarachnoid hemorrhage (OR 0.82, 95% CI 0.67–1.00) but did not increase that of ICH (OR 1.06, 95% CI 0.93–1.21). In addition, patients who have taken aspirin for longer than 3 years may be protected from subarachnoid hemorrhage (OR 0.63, 95% CI 0.45–0.90) compared to those who have not taken aspirin.^23^ Furthermore, results from a large‐scale observational study in the United States (Atherosclerosis Risk in Communities, ARIC) involving 15,719 patients with a follow‐up period of more than 20 years revealed that antiplatelet drug users had a lower risk of ICH than nonusers at the most recent follow‐up before ICH (HR 0.53, 95% CI 0.30–0.92), indicating that APT might even reduce the risk of ICH.^24^

The correlation between the risk of ICH and the antithrombotic benefit of APT has to be further validated (Table 1). Some studies demonstrated that patients with low thrombotic event risk (e.g., healthy individuals aged <50 years) might not be suggested to start APT for primary antithrombotic prevention.^25^, ^26^ The Physicians' Health Study, a randomized, double‐blind, placebo‐controlled trial, found that aspirin only reduced the incidence of myocardial infarction in healthy individuals aged >50 years.^25^ Another study found that prophylactic aspirin use did not reduce the rate of nonfatal strokes or myocardial infarction in healthy individuals and that the incidence of disabling strokes was higher in the aspirin treatment group.^26^ The factors that may increase the risk of ICH in patients receiving APT treatment should be evaluated in advance. It is well known that patients with hypertension have an increased risk of hemorrhagic stroke.^27^ Furthermore, Puddey's review noted that a negative relationship between cholesterol levels and brain bleeding risk has been observed in multiple studies.^28^ Furthermore, according to an epidemiological survey, the incidence of ICH varies by ethnic groups, with the incidence of ICH per 100,000 person‐years being 51.8 for Asians, 24.2 for whites, 22.9 for blacks, and 19.6 for Hispanics.^29^ To summarize, patients with a high risk of bleeding (e.g., hypertensive patients with a low level of cholesterol or Asians) might require close monitoring when taking antiplatelet drugs.

**TABLE 1:** Effect of prior APT on ICH occurrence.

Author	Year of publication	Drug	Patient(age: years old, male sex %)	Area	Increased incidence of ICH
He^14^	1998	aspirin	53–75; 47–86%	USA, Sweden, UK, Europe, Canada	Yes (95% CI 5–20; *p* < 0.001)
Hald^22^	2021	aspirin, clopidogrel	72.8; 52.3%	Denmark	Yes (*p* < 0.001)
García‐Rodríguez^23^	2013	aspirin, clopidogrel, dipyridamole	70.8; 51.1%	Spain	No (OR 1.06, 95% CI 0.93–1.21)
Palacio^32^	2015	clopidogrel+aspirin	NA	Canada	No (OR 1.12, 95% CI 0.86–1.46)
Sharma^24^	2021	aspirin, dipyridamole, clopidogrel	54.4; 44%	America	No (HR 0.53, 95% CI 0.30–0.92)

Abbreviations: APT, antiplatelet therapy; CI, confidence interval; HR, hazard ratio; ICH, intracerebral hemorrhage; NA, not mentioned in the article; OR, odds Ratio.

### 2.2 Dose‐dependent relationship between APT and the incidence of ICH

According to the subgroup analysis results of He's research, there is no dose‐dependent relationship between aspirin use and ICH risk.^14^ Taylor et al. compared the bleeding risk of patients with low‐dose (81 or 325 mg) or high‐dose (650 or 1300 mg) aspirin administration after endarterectomy; the results showed that the high‐dose aspirin group did not exhibit a higher risk of hemorrhage stroke (95% CI 0.77–3.68, *p* = 0.18).^15^ As previously stated, dual APT is recommended during the first 21 days after an acute cerebral infarction or transient ischemic attacks. It is unknown whether the risk of ICH is increased more by dual APT than by mono‐APT. In previous studies, the use of dual APT in patients after stenting was linked to an increased risk of bleeding and a 3‐ to 5‐fold increased risk of death; however, the risk or mortality associated with ICH was not reported.^30^, ^31^ A meta‐analysis reported that in the general population, dual APT was associated with a 23% reduction in the incidence of ischemic stroke attacks (OR 0.77, 95% CI 0.70–0.85) and a 12% (nonsignificant) increase in the risk of ICH (OR 1.12, 95% CI 0.86–1.46) compared with aspirin alone. However, dual APT was associated with a 40% increase in the incidence of major hemorrhage (OR 1.40, 95% CI 1.26–1.55).^32^

Overall, no evidence of a dose‐dependent relationship between APT and the incidence of ICH was noted. Dual APT may thereby reduce the probability of subsequent ischemic cardiovascular and cerebrovascular events, although this beneficial effect may be counterbalanced by a higher risk of significant bleeding.

### 2.3 Association between APT and the incidence of ICH in patients with cerebral microbleeds (CMBs)

Small, normal, or almost normal chronic blood product lesions in the brain tissue are known as CMBs, and they are typically detected using blood‐sensitive magnetic resonance sequences (e.g., T2*‐weighted GRE sequences or susceptibility‐weighted imaging sequences).^33^ The incidence of CMBs was as high as 11.1%–23.5% in a survey of elderly individuals (age > 60 years) living in a community.^34^, ^35^ Similar to spontaneous ICH, the location of CMBs has been reported to indicate different disease causes, such as those located in the cerebral lobe, which may be associated with cerebral amyloid angiopathy, and those located in the deep basal ganglia, which may be associated with hypertension and arteriosclerosis.^35^ CMBs are also found in healthy elderly individuals.^33^ Currently, hypertension is thought to be the best predictor of CMBs, with OR values of 2.3 and 3.9 in healthy adults and stroke patients, respectively.^36^ Other risk factors for ICH, such as age^37^ and low serum cholesterol levels,^35^ have also been linked to the occurrence and extent of CMBs. Several studies have discovered that individuals with CMBs may experience more ICH events and recurrent hemorrhages than those without CMBs.^38^, ^39^, ^40^ Gregoire et al. reported that the risk of ICH increases with an increased number of CMBs (adjusted OR 1.33 per additional microbleed, 95% CI 1.06–1.66, *p* = 0.013).^41^

Whether APT increases the risk of ICH in patients with CMBs remains unclear. Naganuma et al. found the use of APT did not increase the risk of ICH significantly in hemodialysis patients with CMBs.^42^ According to Lovelock's meta‐analysis, the presence of CMBs increases the risk of ICH in antiplatelet drug users compared with nonusers (OR 12.1, 95% CI 3.4–42.5, *p* < 0.001); this implies that when CMBs are present, users of antiplatelet drugs are more likely to develop ICH.^43^ Similarly, Qiu et al. found that among patients receiving APT, those with CMBs had a higher risk of developing ICH than those without CMBs (OR 3.40, 95% CI 2.00–5.78, *p* = 0.000).^44^

According to a recent study, APT may also increase the risk of CMBs. Previous studies have reported that antithrombotic drug use is a risk factor for CMBs.^45^ Qiu et al. found that antiplatelet medication users had more CMBs than nonusers (OR 1.21, 95% CI 1.07–1.36, *p* = 0.002). In addition, antiplatelet drug use was associated with lobar CMBs (OR 1.45, 95% CI 1.15–1.84, *p* = 0.002), whereas no association was observed with deep CMBs (OR 1.37, 95% CI 0.98–1.90, *p* = 0.062).^44^ In the future, additional research is required to investigate the relationship between APT and different sites of CMBs. Taken together, we herein summarized the findings of many studies to elucidate the effect of APT on the incidence of ICH in patients with CMBs. When starting APT in patients with multiple CMB, clinicians should be cautious of the risk of ICH. Furthermore, APT should be initiated with caution in patients who have risk factors for bleeding (e.g., multiple large CMB, and lobar CMB).^41^

## 3 EFFECT OF APT ON ICH OUTCOME

### 3.1 Relationship between prior APT and ICH mortality

Previous studies have revealed that ICH patients with hematoma volumes greater than 30 mL, intraventricular hemorrhage (IVH) or hematoma enlargement was associated with higher mortality and disability rates.^46^, ^47^, ^48^, ^49^ However, the impact of prior APT on mortality and prognosis in ICH patients remains unclear. According to Thompson's systematic study, individuals with spontaneous ICH and prior APT had a higher mortality rate (OR 1.27, 95% CI 1.10–1.47), although there was no significant correlation between these factors and poor functional outcomes (defined as modified Rankin Scale (mRS) score of >2) (OR 1.10, 95% CI 0.93–1.29).^18^ The authors proposed that APT may result in increased mortality in patients with ICH by increasing hematoma volume via an antithrombotic effect. However, because Thompson's study lacks data on the follow‐up hematoma volume, this conjecture cannot be proven.^18^ In addition, Roquer et al. demonstrated that compared with ICH patients without prior APT, adjusted odds of very early (≤24 h) mortality and 3‐month mortality were 2.55 (*p* = 0.004) and 1.56 (*p* = 0.046), respectively, in ICH patients with prior APT.^46^ Moreover, the higher super‐early mortality (within 24 h) in ICH patients receiving prior APT could be because these patients are typically older, have poorer functional status, and have more vascular disease risk factors than those who do not receive APT. However, APT did not affect mortality from 24 h to 3 months. Therefore, APT's significant effect on 3‐month mortality could be a subrogated effect on very early mortality.^46^ Apostolaki‐Hansson et al. compared the prognosis of patients with antiplatelet‐associated ICH (*n* = 3637), anticoagulant‐associated ICH (*n* = 2300), and non‐antithrombotic‐associated ICH (*n* = 7354) in a large observational study APT was found to be significantly associated with both 90‐day mortality (HR 1.23, 95% CI 1.14–1.33) and early mortality (HR 1.32, 95% CI 1.13–1.54) in ICH patients but not with functional dependency (mRS score 3–5; OR 1.07, 95% CI 0.92–1.24).^50^ A review of data from Spain further demonstrated that APT was significantly correlated with in‐hospital mortality of ICH patients.^51^ Overall, these studies indicated that prior APT might affect mortality in ICH patients.

However, some other studies did not show a significant association between APT and ICH outcome. Foerch et al. reported that after adjusting for age and pre‐disease status, APT did not affect in‐hospital mortality (OR 1.12, *p* = 0.490) and poor prognosis at hospital discharge (defined as mRS>2, OR 0.97, *p* = 0.830) in patients with ICH.^52^ According to a database analysis conducted by the Chinese Stroke Center Alliance (CSCA), there was no increase in the mortality rate of patients with prior APT.^53^

As previously stated, findings regarding the effects of APT on the mortality and prognosis of ICH patients remain controversial (Table 2), which may be because most studies were designed retrospectively and the study samples were small with inconsistent inclusion and exclusion criteria.^54^, ^55^ Moreover, the different definitions of outcomes, including mortality, in different studies (some are mortality within 24 h of admission, whereas others reported in‐hospital mortality) would lead to an incorrect estimation of the effect of APT on the ICH‐associated mortality rate.^46^, ^52^

**TABLE 2:** Effect of prior APT on ICH outcome.

Author	Year of publication	Drug	Patient(age: years old, male sex %)	Area	Increased ICH mortality or HE
Roquer^46^	2017	aspirin, clopidogrel	80; 52.4%	Spain	Prior APT caused increased 3‐month mortality (OR 1.56, 95% CI 1.01–2.42, *p* = 0.046)
Al‐Shahi^64^	2018	NA	79; 62%	UK	Prior APT caused increased HE(OR 1.68, 95% CI 1.06–2.66, *p* = 0.026)
Apostolaki‐Hansson^50^	2021	Aspirin, clopidogrel, dipyridamole, ticagrelor	78.9; 51.9%	Sweden	Prior APT caused increased 90‐day mortality (HR 1.23, 95% CI 1.14–1.33)
Law^65^	2021	NA	75.7; 59.9%	UK	Prior APT caused increased 90‐day mortality (OR 1.63, 95% CI 1.25–2.11, *p* < 0.001) and HE (OR 1.28, 95% CI 1.01–1.63, *p* = 0.046)
Sansing^67^	2009	Aspirin, clopidogrel, dipyridamole, triflusal, indobufen	71; 77.1%	USA	Prior APT showed no affect in HE (*p* = 0.16)
Sprugel^57^	2018	NA	76; 56.6%	Germany	Prior APT showed no affect in mortality (*p* = 0.67)
Murthy^68^	2020	NA	66.5; 66.5%	USA	Prior APT showed no affect in mortality and HE
Liu^53^	2021	Aspirin, clopidogrel, dipyridamole, ticagrelor, cilostazol	67; 65%	China	Prior APT showed no affect in in‐hospital mortality (*p* = 0.2372)

Abbreviations: APT, antiplatelet therapy; CI, confidence interval; HE, hematoma expansion; HR, hazard ratio; ICH, intracerebral hemorrhage; NA, not mentioned in the article; OR, odds Ratio.

Vitamin K antagonist‐related ICH is defined as brain hemorrhage caused by effective anticoagulation therapy with vitamin K antagonist, as evidenced by an international normalized ratio of >1.5 on admission.^56^ Some patients are administered anticoagulants and antiplatelet drugs sequentially or in combination. In a study on patients with vitamin K antagonist‐related ICH, patients with prior APT had greater hematoma volume (OR 1.80, 95% CI 1.20–2.70, *p* = 0.005), poorer functional outcomes (mRS score 0–3; 23.8% vs. 31.9%; *p* = 0.030), and a higher mortality (51.0% vs. 40.4%; *p* = 0.009) than those without APT. This could be because the addition of antiplatelet agents to the treatment regimen of vitamin K antagonist users has a greater anti‐hemostatic effect. The hematoma volume in vitamin K antagonist‐related ICH patients receiving APT was larger in that study compared with those who did not receive APT, which may also be a cause of high mortality and poor prognosis. Therefore, the risk of ICH should be carefully considered when vitamin K antagonist is administered in combination with antiplatelet medications. If the combination is required, it is preferable to limit its use to a short period.^57^

### 3.2 Relationship between prior APT and the incidence of hematoma enlargement (HE) in ICH patients

HE is defined as an increase in hematoma volume of >33% or > 12.5 mL from the initial hematoma volume on a steady‐state computed tomography (CT) scan.^58^ Warfarin use, a short time from onset to admission, an irregular hematoma shape, a low level of consciousness, alcohol dependence, and low fibrinogen levels have all been identified as risk factors for HE.^59^, ^60^ Furthermore, in ICH patients, HE is an independent predictor of mortality and unsatisfactory functional prognosis.^61^ Therefore, it is important to validate whether APT affects HE in ICH patients.

According to some reports,^62^, ^63^ prior APT may increase the risk of HE in ICH patients. In a study on the effects of APT on ICH patients during the acute phase, Toyoda et al. discovered that APT was a reliable indicator of acute HE (OR 7.67, 95% CI 1.62–36.4), emergent surgical evacuation of hematoma (OR 3.10, 95% CI 1.18–8.15), and acute death (OR 3.10, 95% CI 1.18–8.15).^63^ In that study, APT was found to be an independent predictor of HE in ICH patients, which could be due to long‐term APT inhibiting platelet function, resulting in active bleeding.^63^ A meta‐analysis conducted by Al‐Shahi et al. demonstrated that prior APT was a potent independent predictor of HE (OR 1.68, 95% CI 1.06–2.66, *p* = 0.026).^64^ In ICH patients, prior APT was linked to a higher risk of HE (adjusted OR 1.28, 95% CI 1.01–1.63), worsening of functional outcome transition (adjusted OR 1.58, 95% CI 1.32–1.91), and higher 90‐day mortality (adjusted OR 1.63, 95% CI 1.25–2.11) according to a subgroup analysis of Tranexamic Acid for Intracerebral Hemorrhage‐2 (TICH‐2) trial. APT was found to increase HE in ICH patients in that study, possibly because antiplatelet drugs disrupted hemostasis. Meanwhile, other factors should also be considered. For example, people who receive APT are older and exhibit increased leukoaraiosis compared with those who do not receive APT.^65^

Tranexamic acid is an antifibrinolytic medication that has been shown to reduce the incidence of HE in patients with spontaneous ICH but not to improve functional outcomes.^66^ It was discovered that regardless of the TICH‐2 subgroup, APT and tranexamic acid therapy could reduce hematoma volume (adjusted OR 0.61, 95% CI 0.41–0.91), implying that APT did not interfere with tranexamic acid's inhibitory effect on HE in ICH patients.^65^ Tranexamic acid significantly reduced HE in patients with prior APT (adjusted OR 0.61; 95% CI, 0.41–0.91) compared with those without prior APT (adjusted OR 0.81, 95% CI 0.64–1.03). In addition to inhibiting platelet aggregation, antiplatelet agents have been shown to increase vulnerability to fibrinolysis by inhibiting acetyl‐lysine residues in fibrinogen, which may play a role in HE. Tranexamic acid may inhibit the breakdown of fibrin clots, improving fibrin solubility in ICH patients with prior APT to prevent HE.^65^ However, further research is needed to elucidate the effect of tranexamic acid on ICH patients with prior APT.

Other studies, however, failed to identify a relationship between HE and APT. Sansing's study revealed that in ICH patients, prior APT was not related to the degree of hematoma present at entry, HE, edema volume, or edema expansion. Furthermore, multivariate analysis showed that prior APT was not significantly associated with any degree of HE (risk ratio [RR] 0.85, upper limit of confidence interval [UCI] 1.03, *p* = 0.16), hematoma volume increased over 33% (RR 0.77, UCI 1.18, *p* = 0.32), and clinical outcome at 90 days (OR 0.67, 95% CI 0.39–1.14, *p* = 0.14).^67^ The enrolled subjects were strictly screened in that study, and the first CT time and CT review time of admitted patients were defined consistently.^67^ In a large diverse cohort study, Murthy et al. investigated the impact of prior APT on HE and functional outcomes in ICH patients. Prior APT was found to be unrelated to HE (OR 0.97, 95% CI 0.60–1.57), severe disability or death (OR 1.05, 95% CI 0.61–1.63), all‐cause mortality (OR 0.89, 95% CI 0.47–1.85), admission hematoma volume (beta −0.17, SE 0.09, *p* = 0.07), or changes in mRS score (*p* = 0.43). In patients with lobar hemorrhage, subgroup analysis revealed a possible correlation between prior APT and admission hematoma volume (beta 0.25, SE 0.12, *p* = 0.03). Prior APT did not affect HE and other functional outcomes in ICH patients, regardless of the location of the hematoma.^68^ Moreover the CT scan time was strictly defined in that study, as was the APT scheme before ICH, which may have been overlooked in other studies.^68^

Based on the abovementioned findings, the effect of APT on HE in ICH patients remains debatable (Table 2). Enlargement of the hematoma typically occurs 6 h after the onset of ICH, with rapid expansion occurring within 3 h.^69^ Different HE assessment times may result in inconsistent results across studies. There was no information provided in Toyoda's study on the time from onset to first CT.^63^ Murthy's study included a certain percentage of patients with onset times greater than 3 h. Because it cannot be ruled out that some cases of HE occurred before the first CT scan, the incidence of HE may be incorrectly estimated.^67^ Furthermore, Murthy's study only included patients with moderate‐to‐small hematoma volumes (median 13.7 mL in the antiplatelet agent group, median 15.7 mL in the non‐antiplatelet agent group); thus, some patients with very large hematomas caused by or exacerbated by APT may have been excluded.^67^ In conclusion, the impact of APT on HE in ICH patients remains unknown because of the differences in study designs. Factors such as the time from the onset to the first CT, the site of hematoma, and the initial hematoma volume should be considered in future clinical studies.

### 3.3 Relationship between different type of antiplatelet drugs and prognosis in ICH patients

Antiplatelet drugs can inhibit platelet aggregation by targeting different sites. Based on their molecular mechanisms, antiplatelet drugs can be categorized as cyclooxygenase inhibitors (COX‐I, e.g., aspirin), adenosine diphosphate receptor inhibitors (ADP‐I, e.g., clopidogrel or ticlopidine), and phosphodiesterase inhibitor (PDE‐I, e.g., dipyridamole or cilostazol).^70^ Whether prior therapy with different antiplatelet drugs exhibits distinct prognosis in ICH patients remains unknown. Using data from the National Health Insurance Research Database, Liu et al. compared the effects of different antiplatelet drugs on mortality in ICH patients. The results showed that prior ADP‐I (OR 1.49, 95% CI 1.24–1.79) and COX‐I (OR 1.17, 95% CI 1.09–1.25) treatments were significantly associated with an increased risk of in‐hospital mortality in ICH patients, whereas there was no discernible difference in in‐hospital mortality between PDE‐I subgroup patients and ICH patients without APT (OR 1.03, 95% CI 0.91–1.16).^70^ This implies that the intricate mechanisms of antiplatelet medications may produce various results in ICH patients, and PDE‐1 may be a more attractive candidate for patients at risk of developing ICH. However, a study from Denmark revealed that a history of treatment with clopidogrel (an ADP‐I) had no appreciable impact on mortality in ICH patients; however, ICH patients receiving prehospital aspirin (a COX‐I) exhibited higher 30‐day mortality than those not receiving APT.^71^ Therefore, the prognosis of ICH patients is affected differently by different antiplatelet medications. Further research is needed to determine which antiplatelet medication type has greater safety while exerting an acceptable antiplatelet aggregation impact. It is advisable to choose a medicine with a higher safety rating for some individuals with a high risk of ICH (such as elderly patients or patients with uncontrollable hypertension).

### 3.4 Prognostic effects of platelet transfusion in ICH patients with prior APT

Naidech et al. revealed that decreased platelet function in patients with spontaneous ICH was associated with HE (*p* < 0.05) and a poor prognosis (defined as mRS score >2 at 3 months, *p* = 0.02).^72^ Thus, platelet infusion may improve the prognosis of ICH patients by improving platelet function.^73^, ^74^ However, there is no evidence suggesting that platelet transfusion lower mortality or improve outcomes in ICH patients.^72^, ^75^, ^76^ Paradoxically, a study showed that platelet transfusion may potentially be detrimental to patients with spontaneous ICH when no emergency surgery indications exist.^77^ Magid‐Bernstein et al. proposed that transfusion of incompatible platelets may be the reason for this negative outcome. The results showed that transfusion of ABO‐incompatible platelets might worsen prognosis (defined as mRS score 4–6; adjusted OR 3.61, 95% CI 0.97–13.42, *p* = 0.06) and increase mortality rates in ICH patients (adjusted OR 2.59, 95% CI 1.00–6.73, *p* = 0.05).^78^ More research on the fundamental systems is required. The hemostatic effect of desmopressin and platelet transfusion on hematoma volume and functional outcomes in ICH patients receiving APT were studied by Mengel et al. The findings revealed that desmopressin and platelet transfusion did not increase the risk of HE. Moreover, secondary IVH, hydrocephalus, and thromboembolic events did not occur at significantly different rates, and the treatment group did not display better 3‐month functional outcomes (defined as mRS score 0–3) than the control group.^79^ Li et al. investigated the impact of platelet transfusion on hypertensive basal ganglia ICH patients undergoing craniotomy. The findings demonstrated that in the subgroup of patients with reduced platelet function, platelet transfusion reduced postoperative recurrence hemorrhage.^80^ In the future, more extensive research is required owing to the limited research sample size to support the conclusion (Table 3). The 2022 AHA/ASA guidelines do not recommend routine platelet transfusion in individuals with spontaneous ICH in light of the aforementioned research findings.^81^

**TABLE 3:** Prognostic effects of platelet transfusion in ICH patients with prior APT.

Author	Year of publication	Drug	Patient(median age: years old; male sex %)	Platelet infusion time and dose	Area	Outcome
Li^80^	2013	Aspirin	NA	Within 48 h of hospitalization; 2.5*10^11 platelets*2	China	Platelet transfusion caused decreased 180 days mortality (*P* < 0.005)
Suzuki^74^	2014	Aspirin, clopidogrel, and ticlopidine	74; 71.2%	NA	Japan	Platelet transfusion caused decreased 90 day‐mortality(*p* = 0.008)
Baharoglu^77^	2016	Aspirin, carbasalate calcium, clopidogrel, dipyridamole	74.2; 57%	within 6 h of ICH onset and within 90 min of diagnostic brain imaging; NA	UK, France	Platelet transfusion caused increased poor prognosis at 3 months (*p* < 0.05)
Mengel^79^	2020	Aspirin and/or P2Y12 inhibitors	73; 63.6%	within 60 min after diagnostic CT; 2 U platelet concentrates	UK	Vasopressin + platelet transfusion showed no affect in 3‐month functional outcome (*p* = 0.309)
Magid‐Bernstein^78^	2021	NA	65; 57.6%	within 24 h of hospitalization; single platelet transfusion (>3.0*10^11 platelets)	USA	ABO‐incompatible platelet transfusion increased 3 month‐mortality (*p* = 0.05) and poor mRS (*p* = 0.06)

Abbreviations: CI, confidence interval; h, hour; ICH, intracerebral hemorrhage; mRS, modified Rankin Scale; NA, not mentioned in the article; OR, odds Ratio.

## 4 REINITIATE APT POST‐ICH

### 4.1 Whether APT be restarted after ICH?

The use of APT after ICH remains debatable in clinical settings. APT following ICH may lower the risk of thromboembolism without increasing the risk of recurrent ICH according to some observational studies.^82^, ^83^, ^84^ When determining whether to restart APT, the risk of recurrent bleeding and ischemic attack risk should be considered. The Restart or Stop Antithrombotics Randomized Trial (RESTART) is the first study to investigate the safety and efficacy of APT after ICH.^85^ This study included 537 ICH patients (268 who started APT and 269 who did not). The average follow‐up period was 2 years, and the median time to restart APT is 76 days (interquartile range 29–146) after the commencement of ICH. The results demonstrated that 12 (4%) of the 268 patients in the APT group had recurrent ICH compared with 23 (9%) of the 268 patients in the avoid APT group who experienced recurrent ICH; nevertheless, there was no significant difference between the two groups (adjusted HR 0.51, 95% CI 0.25–1.03, *p* = 0.06).^85^ These findings imply that secondary avoidance of ischemia episodes in ICH patients with restarted APT is likely advantageous to balance the risk of recurrent ICH, which is probably negligible. APT was not significantly associated with recurrent ICH and serious macrovascular events (e.g., nonfatal myocardial infarction, nonfatal stroke [ischemia, hemorrhage, unexplained cause], vascular death) based on the results of the subsequent extended follow‐up period (2–5 years) analysis.^86^ In a subgroup analysis of RESTART, it was evaluated whether diffusion‐weighted imaging (DWI) lesions affect the recurrence rate of stroke in ICH patients following APT resumption. The results showed that the presence of lesions on DWI was associated with all types (ICH and ischemic) of stroke (adjusted HR 2.2, 95% CI 1.1–4.2) and recurrent ICH (adjusted HR 4.8, 95% CI 1.8–13.2) in ICH patients but not with recurrent ischemic stroke (adjusted HR 0.9, 95% CI 0.3–2.5). This result suggests that DWI lesions should be considered when assessing whether restarting APT would increase the likelihood of recurrent ICH.^87^ Averaging 14.8 months after the onset of ICH, Flynn et al. followed up on 417 ICH patients, of whom 28.8% had been prescribed antiplatelet medications. According to risk factor analysis, the HR of antiplatelet medicine exposure for ischemic stroke and recurrent ICH were 1.07 (95% CI 0.24–4.84) and 0.23 (95% CI 0.03–1.68), respectively. Based on these findings, antiplatelet medication use does not appear to be a substantial risk factor for recurrent ICH.^88^ Moon et al. followed up on ICH survivors with clinical events for 12 years. The results revealed that restarting APT after ICH reduced the occurrence of primary outcome events (nonfatal ICH recurrence, nonfatal ischemic stroke, nonfatal myocardial infarctions; adjusted HR 0.743, 95% CI 0.578–0.956) and all‐cause mortality (adjusted HR 0.740, 95% CI 0.552–0.991) without increasing the risk of recurrent ICH, especially in patients without prior APT.^89^ Weimar et al.^90^ and Viswanathan et al.^91^ further supported this finding, demonstrating that the usage of antiplatelet medications following ICH treatment in patients was not linked to recurrent ICH. Furthermore, a comprehensive review that included data from nine randomized trials revealed that the overall OR of recurrent ICH in patients taking antithrombotic treatment was 1.00 (95% CI 0.73–1.37, *p* = 1).^92^ The dependability and safety of using antiplatelet medications following ICH, however, were not proven. In a former APT‐treated cohort, Jung et al. investigated the impact of restarting APT after ICH on recurrent stroke occurrences. The results showed that restarting APT not only reduced ischemia vascular events (HR 0.240, 95% CI 0.077–0.750, *p* = 0.014) but also decreased recurrent ICH events (HR 0.180, 95% CI 0.055–0.586, *p* = 0.004). This shows that restarting APT after ICH to prevent thromboembolic events in individuals with prior APT is safe.^93^

Murthy et al. followed up on the functional outcome and mortality of ICH patients with restarted APT in multiple centers, and the average interval time of oral antiplatelet drug use was 7–39 days after ICH onset. Oral antiplatelet drug ues after ICH was not significantly associated with mortality (HR 0.85, 95% CI 0.66–1.09) or major disability (HR 0.83, 95% CI 0.59–1.16) compared with those without APT. Subgroup analyses according to hematoma location (lobar or deep) revealed similar results. This implies that restarting APT again following ICH is safe, irrespective of the location of the bleeding.^94^ Additionally, Chen's cohort demonstrated^95^ that commencing APT after ICH was not related to functional outcomes at 3 months (defined as mRS score 0–2).

In summary, most of the current findings suggest that regardless of whether APT was administered before ICH, restarting APT after ICH can reduce the incidence of ischemic events without increasing the rate of recurrent ICH. Meanwhile, APT after ICH may not result in higher mortality and a worse functional prognosis (Table 4). The Antiplatelet Secondary Prevention International Randomised Trial After INtracerebral haemorrhaGe (ASPIRING) is an ongoing multicenter, prospective, randomized, blind outcome clinical trial. ICH patients will be included to evaluate the effect of antiplatelet monotherapy on severe vascular events. A total of 120 patients are planned to be enrolled in this study who will be randomly classified into two groups—starting antiplatelet monotherapy and avoiding antiplatelet monotherapy—in 30 hospitals in China, Australia, and New Zealand (ASPIRING, NCT04522102). This study is expected to provide a high level of evidence for APT initiation in ICH patients.

**TABLE 4:** Safety assessment of restarting APT at different time points after ICH.

Author	Year of publication	Drug	Time between ICH and restart APT	Patient(age: years old, male sex %)	Area	Increased incidence of recurrent ICH
Keir^92^	2002	Aspirin, ticlopidine, dipyridamole	8d‐3 m	NA	UK	No
Viswanathan^91^	2006	NA	NA	NA	US	No [lobar hemorrhage (*p* = 0.73) or deep hemorrhage (*p* = 0.88)]
Flynn^88^	2010	NA	Median: 14.8 m	70.3; 48.3%	UK	No
Weimar^90^	2011	NA	NA	NA	German	No (*p* = 0.173)
Chong^102^	2012	Aspirin, clopidogrel	NA	59.2; 62.3%	China	No (*p* = 0.70)
RESTART^85^	2019	Aspirin, dipyridamole, or clopidogrel,	Median: 7 d	77; 65%	UK	No (*p* = 0.06)
Moon^89^	2021	acetylsalicylic acid, clopidogrel, cilostazol, ticagrelor and ticlopidine	median:0.96y	NA	South Korean	No
Jung^93^	2022	NA	within 6 m	66.7; 47.5%	Korea	No (*p* = 0.004)

Abbreviations: APT, antiplatelet therapy; CI, confidence interval; d, day; HR, hazard ratio; ICH, intracerebral hemorrhage; m, mouth; NA, not mentioned in the article; OR, odds Ratio; y, year.

### 4.2 What should be taken into consideration when restarting APT post ICH?

Some researchers hypothesized that ICH patients with various bleeding sites would react to antiplatelet medications differently. Thus, the location of hemorrhage is an important factor to consider when administering antiplatelet drugs to ICH survivors. After controlling for fundamental clinical markers, Biffi et al. discovered that aspirin use was an independent risk factor for recurrent ICH in primary lobar ICH patients (HR 3.95, 95% CI 1.6–8.3, *p* = 0.021).^96^ However, owing to the scarcity of data, this study may have an overfitting bias. Other studies, however, found no link between APT and recurrent ICH. According to one study, antiplatelet drugs were administered to 22% of ICH survivors to prevent ischemic heart disease (lobar hemorrhage 27/127, deep hemorrhage 19/80). In that study, antiplatelet drugs were not found to be associated with recurrent ICH in lobar hemorrhage (HR 0.8, 95% CI 0.3 to 2.3, *p* = 0.73) or deep hemorrhage survivors (HR 1.2, 95% CI 0.1 to 14.3, *p* = 0.88).^91^ Furthermore, it has been proposed that the presence of CMBs may influence the decision to restart APT following ICH. According to Wilson's review, rebleeding events are significantly positively associated with the number of CMBs in patients with lobar hemorrhage, and antithrombotic drugs should be avoided unless there is a strong antithrombotic indication.^97^

Restarting APT after ICH in some individuals with concomitant comorbidities is also important to consider. Atrial fibrillation (AF) and spontaneous ICH are occasionally observed in clinical settings. Rocher et al. examined whether the presence of AF influenced the outcome of ICH patients who had previously undergone APT. The findings indicated that in‐hospital and 3‐month mortality was higher in ICH patients with AF than in those without AF. AF did not affect mortality after excluding the presence of APT (OR 1.45, 95% CI 0.74–2.85, *p* = 0.284).^98^ It is unknown, however, whether restarting APT treatment affects the prognosis of ICH patients with AF. The distribution of antiplatelet medication use following ICH in AF and non‐AF individuals was compared by Pennlert et al. The proportion of AF patients with antiplatelet drug use was 36.6% within 6 months and 43.6% within 1 year. In the population without AF, the corresponding proportions were 13.8% and 17.5%, respectively. However, because the ischemic events and rebleeding events in this trial were not followed‐up on, further information is required to determine the safety and effectiveness of APT after ICH.^99^ According to Lin's findings, patients with AF who restarted APT following an ICH did not show a reduction in ischemic events (HR 1.13, 95% CI 0.81–1.56); instead, it even increased the risk of recurrent ICH (HR 1.81, 95% CI 1.07–3.04).^100^ This shows that antiplatelet medications may have minimal benefit for the ICH patients with AF and even lead to a higher risk of rebleeding after restarting APT.

Cho et al. investigated the effectiveness and safety of warfarin and antiplatelet medication use in ICH patients with a left ventricular assist devices (LVAD). In all LVAD patients, the median time to restart APT is 8 days after ICH onset. Patients receiving antiplatelet drugs alone experienced higher thrombotic events than those receiving either warfarin alone or both warfarin and antiplatelets (1.37 vs. 0.14 events/patient‐year [EPPY], *p* = 0.08). Nonfatal hemorrhage events rates were 0.34 EPPY in the only warfarin or both warfarin and antiplatelet group and 0 EPPY in the only antiplatelet group (*p* = 0.16). No fatal hemorrhage events occurred.^101^ This shows that compared to aspirin alone, the combination of warfarin and aspirin decreased embolic events but did not increase recurrent bleeding episodes.

Chong et al. studied the effect of prescribed aspirin on the rate of recurrent ICH in ICH patients. In total, 440 patients with spontaneous ICH were included, and 56 of them (12.7%) received aspirin after ICH. The results showed that the use of aspirin did not increase the risk of recurrent ICH compared with patients who did not use aspirin (22.7 per 1000 patient‐aspirin years vs. 22.4 per 1000 patient‐years, *p* = 0.70).^102^ Hypertension (HR 2.0, 95% CI 1.06–3.75, *p* = 0.03) and age > 60 years (HR 2.0, 95% CI 1.07–3.85, *p* = 0.03) were found to be independent risk factors for recurrent ICH. In a subgroup analysis of 127 ICH patients with definite antithrombotic indications (coronary artery disease, ischemic stroke, and AF), of whom 56 used aspirin, aspirin use after ICH significantly reduced concomitant vascular events (recurrent ICH, ischemic stroke, and acute coronary syndrome; 52.4 per 1000 patient‐aspirin years, vs. 112.8 per 1000 patient‐years, *p* = 0.04).^102^

In summary, in patients with pre‐existing hypertension, antiplatelet medication use may increase the risk of recurrent ICH; however, this is also the main population that benefits from antiplatelet drugs for the prevention of ischemic events. When restarting APT, additional variables such as bleeding site and concomitant with AF or LVAD should also be taken into consideration.^102^, ^103^

## 5 CONCLUSION

The effect of antiplatelet drugs on the occurrence and development of ICH is still not completely known. Moreover, there is no accepted practice or standard procedure for determining whether and when to restart APT after ICH. A detailed literature search was performed, and relevant findings were summarized in this paper. The risk of ICH related to APT, the impact of prior APT on the characteristics and prognosis of ICH patients, and the safety of restarting APT after ICH were the key topics covered in this review.

When individuals are administered APT, the risk of thrombotic events should be weighted against the risk of bleeding. In general, for some patients’ concomitant with risk factors of thrombotic events, such as hyperlipidemia, hypercoagulability, chronic AF, and severe atherosclerosis, the advantages of antiplatelet medication may outweigh the inherent risk of rebleeding. However, the use of antiplatelet medications for other patients with a higher risk of bleeding, such as elderly individuals and those with multiple CMB and uncontrolled hypertension, should be carefully evaluated and closely monitored. In the future, a quantifiable system for assessing thrombotic risk and bleeding risk will be necessary.

After evaluation, the appropriate time to restart APT for ICH patients should be determined to prevent underlying ischemic stroke events. According to the present study results and expert experience, most patients now restart APT at around 1 week following the onset of ICH. Nevertheless, the precise time to restart APT should be chosen on a case‐by‐case basis as per the patient's risk of embolic events and recurrent bleeding. More compelling evidence‐based medicine evidence is needed in the future.

To summarize, additional high‐quality randomized controlled trials are required in the future to elucidate the relationship between APT and the risk and prognosis of ICH. More specific programs for making judgments about diagnosis and therapy should also be investigated.

## AUTHOR CONTRIBUTIONS

Review program formulation: Yunjie Li, Gaigai Li, Chao Pan; literature screening: Xia Liu; literature reading and data collection: Yunjie Li, Shiling Chen, Jingyi Wang; paper writing: Yunjie Li; language polishing, paper review and editing: Gaigai Li, Zhouping Tang.

## FUNDING INFORMATION

This work was supported by grant 82,201,474 and 82,071,330 from National Natural Science Foundation of China.

## CONFLICT OF INTEREST STATEMENT

The authors declare no conflict of interest.

## DECLARATION

All authors read and approved the final version of the paper and agreed to submit the manuscript to ***CNS Neuroscience & Therapeutics***.

## Data Availability

Data sharing is not applicable to this article as no new data were created or analyzed in this study.
